# Bilingualism can cause enhanced monitoring and occasional delayed responses in a flanker task

**DOI:** 10.1111/ejn.15863

**Published:** 2022-11-23

**Authors:** Roksana Markiewicz, Ali Mazaheri, Andrea Krott

**Affiliations:** ^1^ School of Psychology University of Birmingham Birmingham UK; ^2^ Centre for Human Brain Health University of Birmingham Birmingham UK

**Keywords:** bilingual advantage, conflict monitoring, conflict tasks, N2, P3

## Abstract

Complex cognitive tasks require different stages of processing (i.e. conflict monitoring, attentional resource allocation and stimulus categorisation). Performance differences between bilinguals and monolinguals on conflict tasks can be affected by the balance of these sub‐processes. The current study investigated the effect of bilingualism on these sub‐processes during a conflict task with medium monitoring demand. Behavioural responses and evoked potentials from bilinguals and monolinguals were examined during a flanker task with 25% incongruent trials. Behavioural differences were analysed by means of averaged response times and exponentially modified Gaussian analyses of response time distributions. For evoked potentials, the study focussed on N2 (reflecting conflict monitoring) and P3 responses (reflecting allocation of attentional resources for cognitive control). Bilinguals had significantly longer response distribution tails compared to monolinguals. Bilinguals were shown to have a more pronounced N2 and smaller P3 compared to monolinguals, independent of condition, suggesting a different balance of sub‐processes for the two groups. This suggests that bilinguals were engaged more strongly in monitoring processes, leading to the allocation of fewer attentional resources during stimulus categorisation. Additionally, the P3 amplitudes were negatively related with the length of response distribution tails for bilinguals. These results are consistent with enhanced conflict monitoring in bilinguals that led to reduced engagement of attentional resources for stimulus categorisation. This enhanced conflict monitoring could lead to occasional extremely slow responses. Thus, the bilingual experience appears to impact the balance of cognitive control processes during conflict tasks, which might only be reflected in a minority of responses.

## Graphical abstract

Using evoked‐related potentials and exponentially modified Gaussian analyses, we investigated the effect of bilingualism on the sub‐processes (conflict monitoring and allocation of attentional resources) during a flanker task with medium monitoring demand. We found that bilinguals had significantly longer response distribution tails as well as a more pronounced N2 and smaller P3 components compared to monolinguals.

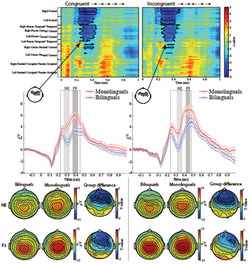

**Abbreviations**
ACC: anterior cingulate cortex
EEG: electroencephalography
ERP: event‐related potential
Ex‐Gaussian: exponentially modified Gaussian
ICA: independent component analysis
M: mean
PCA: principal component analysis
RT: reaction time
SD: standard deviation
STEM: science, technology, engineering, and mathematics
VIF: variance inflation factor

## 1 INTRODUCTION

Speaking another language leads to changes in brain structure and function, particularly in the networks implicated in cognitive control (Li et al., 2014; Pliatsikas & Luk, 2016). Consequently, monolingual and bilingual speakers differ in performance on tasks of executive control, with many studies finding superior behavioural performance for bilinguals (Bialystok et al., 2004; Costa et al., 2008; Emmorey et al., 2008; Hernández et al., 2010; review in Bialystok et al., 2012; meta‐analyses in Donnelly et al., 2019; Grundy, 2020). However, other studies have found similar performance in the two participant groups or even superior performance in monolinguals (Duñabeitia et al., 2014; Gathercole et al., 2014; Naeem et al., 2018; Paap et al., 2015; review in Nichols et al., 2020; meta‐analysis in Lehtonen et al., 2018). Together, these findings suggest that structural and functional changes brought about by bilingualism lead to differences in cognitive processing, which is not always reflected in a behavioural advantage. What has not been considered much in this debate is that cognitive control tasks contain a number of processes, such as conflict monitoring or engagement of attentional resources for conflict control. Functional and structural changes might affect these processes in different ways. They might make bilinguals more efficient in some and potentially less efficient in other processes involved in a task. The balance of processes involved in a task might therefore potentially explain inconsistencies in the literature. The current study thus investigated the differences in engagement of sub‐processes of cognitive control between monolingual and bilingual speakers and examined how these could lead to behavioural differences.

One common cognitive control task, also used here, is the flanker task (Eriksen & Eriksen, 1974), in which participants need to indicate the direction of the central arrow (the target) in rows of arrows. The flanking arrows point either into the same direction as the target (congruent trials) or into the opposite direction (incongruent trials). The latter condition creates a conflict that needs to be resolved, leading to longer response times and more errors. Although there are some differences between models capturing the processes in the flanker task (see overview in Ridderinkhof et al., 2021), all assume that target and flankers are processed in parallel and activate corresponding responses, leading to competition for incongruent trials (Coles et al., 1985; Eriksen & Eriksen, 1974; Gratton et al., 1988, 1992). Most recently, Ridderinkhof et al. (1995) suggested a dual‐route model. In an automatic ‘direct’ route, both target and flankers prime a response in a bottom‐up fashion, whereas the controlled ‘deliberate’ route handles target selection and identification as well as stimulus–response translation. These two routes are activated in parallel and both prime a response. Responses are merged and might lead to competition. The resolution of the competition requires cognitive control mechanisms.

Important for the present study, processing in the flanker task is under strategic control (Gratton et al., 1992). More specifically, participants can modulate conflict monitoring depending on the mixture of congruent and incongruent trials (Botvinick et al., 2004). In tasks with predominantly incongruent trials, the participants proactively monitor more strongly so that cognitive control can be upregulated (Bugg & Gonthier, 2020). This means they rely more on the controlled ‘deliberate’ processing route (see dual‐route model by Ridderinkhof et al., 1995). In contrast, when trials are predominantly congruent, the participants monitor less for conflict and instead rely more strongly on the automatic ‘direct’ route. The latter leads to faster responses for congruent trials (Botvinick et al., 2004), but because of reduced conflict monitoring, processing of incongruent trials gets more erroneous, effortful and slower (e.g. Gratton et al., 1992).

A comparison of monolingual and bilingual performance in the flanker task suggests that, depending on the task affordances, bilinguals might rely more strongly on the controlled ‘deliberate’ route of processing. They appear to activate their monitoring system more strongly when monitoring demand is higher. For tasks using high levels of either congruent or incongruent trial types, both monolingual and bilingual participants have been shown to perform similarly (Costa et al., 2009). In tasks using predominantly congruent trials, both groups are likely to rely on the direct route of processing, whereas when incongruent trails dominate, both groups rely on the deliberate route of processing (Ridderinkhof et al., 1995). Group differences emerge when the proportion of congruent to incongruent trials falls between 25 and 50% (Abutalebi et al., 2015; Costa et al., 2008, 2009; Emmorey et al., 2008; Zhou & Krott, 2016), that is when optimal performance requires engaging cognitive control processes such as conflict monitoring. In this case, monolinguals potentially avoid constant monitoring because it is too effortful, relying more strongly on the direct route of processing.

Automatic versus controlled processes in a task can be investigated with the means of an exponentially modified Gaussian (ex‐Gaussian) analysis of the reaction time (RT) distribution. This analysis provides the parameters *μ* and τ of the RT distribution, with *μ* capturing general processing speed and τ reflecting a measure of extremeness of slow responses. Previous literature commonly interpreted *μ* as reflecting more strongly stimulus‐driven automatic processes, whereas τ is thought to reflect controlled processes that are attention demanding (see review in Matzke & Wagenmakers, 2009). Specifically in conflict tasks, *μ* is a reflection of response conflict and conflict‐resolving inhibitory control processes (Heathcote et al., 1991; Kane & Engle, 2003; Spieler et al., 2000; Zhou & Krott, 2018), whereas τ mirrors attentional control to maintain the task goal (Epstein et al., 2011; Hervey et al., 2006; Spieler et al., 1996).

In the flanker task, particularly slow responses can occur when participants rely strongly on controlled stimulus processing, thus the deliberate processing route. This could be an over‐reliance on conflict monitoring, meaning a response might occasionally be slowed down because of overactive monitoring processes. It could also be that a controlled process is not very efficient. For instance, fewer attentional resources might be devoted to the cognitive control mechanisms for the deliberate processing route or the merging of direct and deliberate processing routes of the flanker task.

Utilising ex‐Gaussian analyses to investigate the effects of bilingualism in conflict tasks has suggested differences between language groups, particularly in terms of controlled processes. Bilingual speakers have been found to have smaller *μ* and τ (Abutalebi et al., 2015; Calabria et al., 2011) or only smaller τ (Zhou & Krott, 2018). As indicated above, this could mean bilinguals engage in more efficient conflict monitoring. Alternatively or in addition, they might have more efficient cognitive control processes related to the deliberate processing route or the merging of the two processing routes of the conflict task.

Another way of examining differences between monolingual and bilingual participants in functional processing during conflict tasks is to utilise electroencephalography (EEG). Previous EEG research has found differences particularly in the N2 and P3 components. The N2 is a fronto‐central negative deflection that peaks at around 200–300 ms after stimulus onset. A larger N2 amplitude has been associated with enhanced conflict monitoring (Folstein & Van Petten, 2008; Kousaie & Phillips, 2012; Purmann et al., 2011; Yeung & Cohen, 2006) and signalling for stronger cognitive control (Clayson & Larson, 2011). Conditions that involve conflict, for instance, incongruent trials in the flanker task, elicit a more prominent N2 response compared to conditions that are conflict free (i.e. congruent trials) (Danielmeier et al., 2009; Purmann et al., 2011; van Veen & Carter, 2002; Wild‐Wall et al., 2008; Yeung et al., 2004). The N2 appears to be affected by strategic control, because N2 amplitudes are reduced for incongruent trials that follow incongruent trials compared to congruent trials (e.g. Clayson & Larson, 2011; Forster et al., 2011). Previous studies have often reported more pronounced or earlier N2 responses for bilinguals than monolinguals (Chung‐Fat‐Yim et al., 2021; Fernandez et al., 2013, 2014; Kousaie & Phillips, 2012, 2017; Morales et al., 2015; Moreno et al., 2014) and more pronounced N2 amplitudes for bilinguals with higher L2 proficiency (Fernandez et al., 2013, 2014). In line with the interpretation of the N2, larger N2 amplitudes in bilinguals echo greater resource allocation to conflict monitoring and shorter N2 latencies suggest more automatic and faster monitoring. Both differences suggest that bilinguals rely more strongly on earlier processes during a conflict task than monolinguals, reflecting a more proactive approach (Grundy, Anderson, & Bialystok, 2017).

In contrast to the N2, the P3 (also called standard P3 or P3b) is a positive component with centro‐parietal distribution and is associated with attentional resource allocation during stimulus categorisation. Shorter P3 latencies are associated with shorter stimulus evaluation/faster categorisation time (Coles et al., 1985; Kok, 2001; Polich, 2007), and larger amplitudes are proportional to the amount of attentional resources allocated to stimulus processing (Kok, 2001; Wickens et al., 1983) or, more specifically for the flanker task, to the amount of attentional resources needed for cognitive control during stimulus categorisation (Clayson & Larson, 2011). Incongruent trials in the flanker task lead to larger and later P3 responses (Purmann et al., 2011; Wild‐Wall et al., 2008). Similar to the N2, the P3 appears to be under strategic control, being modulated by the mix of congruent/incongruent trials (Purmann et al., 2011) and the nature of preceding trials (Clayson & Larson, 2011).

Differences in P3 between bilinguals and monolinguals in conflict tasks are mixed. Some studies found bilinguals have greater P3 amplitudes (Kousaie & Phillips, 2017; Moreno et al., 2014) and/or shorter P3 latencies than monolinguals (Barac et al., 2016; Kousaie & Phillips, 2012, 2017), whereas others found smaller P3 amplitudes (Botezatu et al., 2021; Coderre et al., 2014; Kousaie & Phillips, 2012). This might be because of variations in the tasks' control demand. Under enhanced control demands, bilinguals show increased N2 components (suggesting enhanced monitoring) and reduced P3 responses (suggesting reduced devotion of attentional resources for cognitive control). This has been found when presenting bilingual participants with flanker trials interleaved with words from two instead of one of their languages (Wu & Thierry, 2013) and when presenting a stop‐signal task after bilinguals used their L2 instead of their L1 (Kałamała et al., 2022), that is after enhanced engagement of inhibitory control processes.

## 2 THE PRESENT STUDY

The aim of the present study was to investigate differences in engagement of sub‐processes of cognitive control between monolingual and bilingual speakers and how these might lead to behavioural differences. For that, we tested young adults on a flanker task. In order to detect group differences in more automatic versus controlled processes, we inspected ex‐Gaussian parameters. To investigate controlled processes between the two participant groups, we studied event‐related potentials (ERPs) during task performance.

Importantly, although cognitive control has been studied utilising both ex‐Gaussian analyses (Abutalebi et al., 2015; Calabria et al., 2011; Tse & Altarriba, 2012; Zhou & Krott, 2018) and ERPs (Botezatu et al., 2021; Kousaie & Phillips, 2012, 2017), to our knowledge, no study to date has combined these analyses and related these measures with each other. Doing so allowed us to investigate whether stronger or weaker engagement of sub‐processes in the task, such as conflict monitoring (N2) and devotion of attention to conflict resolution (P3), is beneficial for more automatic processing (ex‐Gaussian parameter *μ*) or more controlled processing (ex‐Gaussian parameter τ). In addition, it enabled us to investigate whether these relationships might be different for the two participant groups. For that, we analysed whether neural markers that showed differences between groups predicted ex‐Gaussian parameters and whether these predictive relations differed between groups. Yeung and Nieuwenhuis (2009) had found that larger N2 amplitudes in a flanker task were correlated with overall slower responses in a group of young adults. This means that a strong engagement of monitoring processes in bilinguals, reflected in larger N2 amplitudes, could potentially slow down their responses compared to monolinguals, if only occasionally, meaning that a strong engagement of monitoring might be disadvantageous (see above). On the other hand, the need for fewer attentional resources for cognitive control during stimulus categorisation, reflected in decreased P3 amplitudes, might counteract this disadvantage. Since controlled processes such as conflict monitoring or the allocation of attentional resources for conflict control are rather reflected in the *τ* parameter, we might see that the N2 and P3 predict *τ* but not *μ*.

In order to maximise potential strategic differences between monolinguals and bilinguals in conflict monitoring and engagement of attentional resources for cognitive control, we used a version of the flanker task with medium monitoring demand (Costa et al., 2009), that is with 25% incongruent and 75% congruent trials. Because bilinguals have previously found to differ from monolingual participants in terms of overall faster RTs or in terms of smaller congruency effects (for a discussion, see Hilchey & Klein, 2011), we examined both overall language group differences and group differences in congruency effects (i.e. *differences* between incongruent and congruent conditions).

Given previous results, we expected to see stronger bilingual monitoring reflected in enhanced N2 amplitudes. Jiao et al. (2020) showed that increased N2 responses went hand in hand with decreased P3 amplitudes. We therefore might also see decreased P3 amplitudes in bilinguals and thus less subsequent devotion of attentional resources for cognitive control during response selection. We did not have strong predictions about behavioural results. If bilinguals are, as previously suggested, particularly efficient in monitoring in the task, they might outperform monolinguals in terms of accuracy and RTs and they might have shorter response distribution tails. However, with a relatively small percentage of incongruent trials (25%), strong bilingual engagement in monitoring might not be beneficial. In this case, we might not see group differences (Antón et al., 2019; Grundy, Chung‐Fat‐Yim, et al., 2017; Kousaie & Phillips, 2012, 2017; Luk et al., 2010) or even a monolingual advantage.

## 3 METHOD

### 3.1 Participants

Seventy students of the University of Birmingham took part in the study. We recruited participants who fell into one of the two language groups (monolinguals/bilinguals) (see classification criteria in Table 1) based on their responses to a Language History questionnaire (taken from Zhou & Krott, 2018). Sixteen participants were excluded from the analysis because of (a) EEG software malfunction (*N = 7*), (b) excessive EEG artefacts (*N = 5*, rejected trials exceeded 30%), or (c) extremely slow responses (*N = 4*, RT mean was 3 SD above the mean of all participants). This led to 28 monolinguals and 26 bilinguals being included into analyses (see Table 2 for demographic information of participants). All had normal or corrected vision and no neurological impairments. Monolinguals matched their bilingual counterparts in age, *t*(52) = .59, *p* = .556, and fluid intelligence, assessed via Raven's Standard Progressive Matrices (Raven, 1958), *t*(52) = .56, *p* = .580.

**TABLE 1:** Allocation criteria into monolingual and bilingual groups

Monolinguals	Bilinguals
a) No second language before age 7	a) Both L1 and L2 learnt before age 7
b) Language proficiency rating 4 or lower (out of 7) in L2 (or any other language)	b) Language proficiency rating 5 or above (out of 7) in both L1 and L2
c) Use of only L1 on a daily basis	c) Both L1 and L2 used on a daily basis

**TABLE 2:** Demographic information of participants

	Monolinguals	Bilinguals
*N* (male/female)	28 (10/18)	26 (3/23)
Mean age in years (*range, SD*)	20.1 (*18–24, 1.66*)	19.8 (*18–28, 2.04*)
Right handed/left handed	24/4	26/0
Mean age (years) of second language acquisition (*SD*)	10.14 (*2.91*)	3.54 (*2.8*)
Self‐rated proficiency of L1 (*range 0–7*)	6.71 (*6–7*)	6.54 (*5–7*)
Self‐rated proficiency of L2 (*range 0–7*)	2.29 (*1–3*)	6.15 (*5–7*)
Mean Raven's score (*SD*)	50.68 (*5.12*)	49.25 (*9.04*)

*Note*: Handedness was measured using the Handedness questionnaire by Oldfield (1971). Seven of the monolingual participants reported some knowledge of another language.

The participants gave informed consent, which followed the guidelines of the British Psychology Society code of ethics, and the experiment was approved by the Science, Technology, Engineering, and Mathematics (STEM) Ethical Review Committee of the University of Birmingham.

### 3.2 Materials

The flanker paradigm (Eriksen & Eriksen, 1974) was implemented using E‐prime 2.0 (Psychology Software Tools, Pittsburgh, PA). Each trial consisted of five black arrows presented in the centre of a white computer screen, with the central arrow being the target and two flanking arrows on both sides functioning as distractors. Two types of trials were presented: (a) congruent trials (75% of all trials) that comprised of five arrows facing into the same direction and (b) incongruent trials (25% of all trials) that consisted of a central target arrow pointing to the opposite direction than the flankers. The Language History questionnaire (taken from Zhou & Krott, 2018) asked the participants to self‐rate their proficiency of English and any other languages that they learnt or were able to speak. They were also asked to report the age of acquisition of English and any other languages, and their current language use pattern (e.g. using mainly one or using both languages on a daily basis at home/school/social setting).

### 3.3 Procedure

The participants first completed the Language History questionnaire followed by the Handedness questionnaire (Oldfield, 1971), before taking part in the EEG experiment. For the latter, they sat in an isolated room, approximately 55 cm from the computer monitor. Each trial began with the presentation of a fixation cross in the centre of the screen for 800 ms, followed by a stimulus, which stayed on screen for 5000 ms or until response. Each trial was followed by a blank screen for 500 ms. Subjects indicated the direction of the target by pressing corresponding buttons on a Cedrus RB‐834 response pad, which also recorded response times. The task comprised of 10 blocks of 96 trials, with an additional practice block (24 trials), taking approximately 35 min. Stimuli appeared randomly. Finally, the participants completed the Standardised Progressive Matrices assessment (Raven, 1958) for which they were given a 25 min time limit.

### 3.4 EEG recording

Continuous electrophysiological recordings were obtained using a 128‐channel BioSemi Active Two EEG system. BioSemi electrodes are active electrodes with sintered Ag‐AgCI tips/pallets. The electrodes were placed onto the electrode cap by the experimenter using the 5–10 system (Oostenveld & Praamstra, 2001) for electrode positioning. External electrodes placed on the mastoids were used as reference electrodes for offline processing. The signal was amplified with a bandpass of .16–.128 Hz using a BioSemi Active 2 AD‐box, sampled at 512 Hz, and recorded using ActiView version 7.06 software.

## 4 DATA ANALYSIS

### 4.1 Behavioural analysis

We analysed response times (RTs), accuracy, and ex‐Gaussian parameters *μ* (the mean of the Gaussian part of the response distribution) and τ (the mean and standard distribution of the exponential part of the response distribution). We examined group differences using traditional measures (i.e. averaged RTs and accuracy) in order to easily compare the performance of our monolingual and bilingual groups to previous findings (see supporting information and Figure S1 for detailed results). The accuracy rate was the percentage of correct responses. Responses below 200 ms were treated as incorrect. RT and ex‐Gaussian analyses were based on correct responses. Responses greater than 2 SD from each participant's mean were removed from the RT analysis (for a similar approach, see, e.g. Paap & Greenberg, 2013; Zhou & Krott, 2018) but not from the ex‐Gaussian analysis. Ex‐Gaussian parameters *μ* and τ were determined for each participant for each condition (congruent and incongruent) using the QMPE software (Brown & Heathcote, 2003). The exit codes were below 32 for all parameter estimations; therefore, they were considered trustworthy (in line with the QMPE software manual). The average number of search iterations was 7.09. RTs, accuracy, and ex‐Gaussian parameters (*μ* and τ) were analysed each with a mixed 2 (Language Group: monolingual vs. bilingual) × 2 (Condition: congruent vs. incongruent) analysis of variance (see supporting information for RT and accuracy results).

### 4.2 EEG analysis

The EEG data pre‐processing was performed with the means of EEGLAB 14.1.2b (Delorme & Makeig, 2004) and Fieldtrip toolbox 2018‐07‐16 (Oostenveld et al., 2011). EEG data were off‐line filtered with a .1 Hz high‐pass filter and a 30 Hz low‐pass filter, referenced to the average of the mastoid electrodes and epoched from −2 to 2 s locked to the onset of the arrow array. Ocular artefacts in the data were removed based on visual inspection of the scalp distribution and time course of the components, using the independent component analysis (ICA) extended‐algorithm in EEGLAB. Prior to the ICA, a principal component analysis (PCA) was used to reduce dimensionality of the data to 15 components. The average number of removed components per participant was 1.33 (*SD* = .57, min = 1, max = 3), with no difference in the number of components removed between the bilingual (*M* = 1.5, *SD* = .65) and monolingual (*M =* 1.21, *SD* = .5) groups (*t*(52) = −1.81, *p* = .077). The recordings did not show any channels with continuous artefacts or noise throughout the recording session. Instead, excessive temporary artefacts were dealt with and removed on a trial‐by‐trial basis. The Fieldtrip toolbox 2018‐07‐16 (Oostenveld et al., 2011) was used for trial removal. Our criteria for trial rejection were trials with incorrect responses, trials with RT < 200 ms, and trials with RT > 2 SD from the mean of each participant. The average percentage of rejected trials per participant because of behavioural outliers (i.e. reasons outlined above) was 9.41% (SD = 2.39), with no significant difference in the percent of trials removed between the bilingual (*M* = 9.32, *SD* = 1.95) and monolingual (*M* = 9.5, *SD* = 2.77) groups (*t*(52) = .28, *p* > .05). In addition, a semi‐automatic artefact rejection summary method was used, where any trial with signal that exceeded 200 μV per electrode was removed. The average percentage of rejected trials per participant because of artefact rejection was 7.82% (*SD* = 6.46), with no significant difference in the percent of trials removed between the bilingual (*M* = 8.09, *SD* = 6.75) and monolingual (*M* = 7.6, *SD* = 6.31), (*t*(50) = −.28, *p* = .781). The total average percentage of rejected trials per participant included in the analysis was 16.0% (*SD = 6.6*), with no significant difference in the number of trials removed between the bilingual (*M = 15.96%, SD* = 6.8) and monolingual (*M = 16.1%, SD* = 6.45) groups (*t*(52) = .08, *p* = .938). The traditional RT and ERP analyses were conducted using (on average per participant) 620 (*SD* = 95.4) congruent trials and 177.5 (*SD* = 30.5) incongruent trials.

### 4.3 Analysis of ERPs

We averaged the time‐locked EEG activity of all valid trials (see above for removed trials) for each participant and for congruent and incongruent conditions separately. The baseline correction used for the ERP analysis was −200 ms to 0 prior to stimulus (arrow array) onset. We employed a non‐parametric cluster‐based permutation tests (Maris & Oostenveld, 2007) as implemented in Fieldtrip to assess statistical differences of ERP amplitudes in the time window 0–1 s after stimulus onset. These tests are designed to contrast two conditions. We therefore conducted the following analyses: We compared congruent versus incongruent conditions collapsed across participants to check that the interference effect expected in the flanker task was present (results are presented in the supporting information). We checked for group differences in each condition (monolinguals vs. bilinguals in congruent condition; monolinguals vs. bilinguals in incongruent condition), and finally, we compared groups on the flanker effect (i.e. congruency effect in monolinguals vs. congruency effect in bilinguals). More specifically, for each comparison, this approach involved conducting a *t*‐test (dependent samples in the condition contrast, independent samples in the group contrast) for every point in the electrode by time plane and clustering the *t*‐stats in adjacent spatiotemporal points if they exceeded a threshold of *p* < .05 (cluster alpha). The cluster with a Monte Carlo *p*‐value smaller than .025 was identified as significant (simulated by 1000 partitions), thus, showing a significant condition or group difference in amplitude. Probability values for each cluster were obtained by comparing it to a distribution using the Monte Carlo simulation method, in which the group (and equivalent condition) labels were randomly shuffled 1000 times and the maximum sum of the *t*‐stat in the resulting clusters was measured. Here we considered clusters falling in the highest or lowest 2.5th percentile to be significant. The triangulation method was used to define a cluster (i.e. a cluster consisted of at least two significant neighbouring electrodes).

Next, we investigated whether stronger or weaker engagement of sub‐processes in the task, such as conflict monitoring (N2) and devotion of attention to conflict resolution (P3), was beneficial for more automatic processing (ex‐Gaussian parameter *μ*) or more controlled processing (ex‐Gaussian parameter τ). Note that mean N2 and P3 peak amplitudes are assumed to be reflections of a participant's average conflict monitoring and attentional engagement for cognitive control across the task. Ex‐Gaussian parameter *μ* reflects the mean RT of a participant's majority responses, and ex‐Gaussian parameter τ the extremeness of their slow responses. We regressed the ex‐Gaussian parameters *μ* and τ with the mean peak amplitudes of N2 and P3. To see whether these relationships differed for the two participant groups, we added the factor Group (bilinguals, monolinguals) as well as interactions of Group with the neural markers of N2 and P3 (e.g. *μ*_congruent ~ N2 + P3 + Group + N2 * Group + P3 * Group). We conducted separate analyses for the two experimental conditions (congruent and incongruent). In case of a significant interaction, we conducted separate regression analyses for both groups with the predictors N2 and P3. To account for these multiple tests (two ex‐Gaussian parameters and two conditions), we adjusted *α* to .05/4 = *.0125*. Because N2 and P3 amplitudes are highly correlated, we checked collinearity with the means of the variance inflation factor (VIF). In any case where VIF was above 10 for any of the factor, we conducted a second regression analysis after standardising the affected factors (e.g. Iacobucci et al., 2016, 2017). We report VIFs with the final results.

## 5 RESULTS

### 5.1 Behavioural results

**FIGURE 1:**
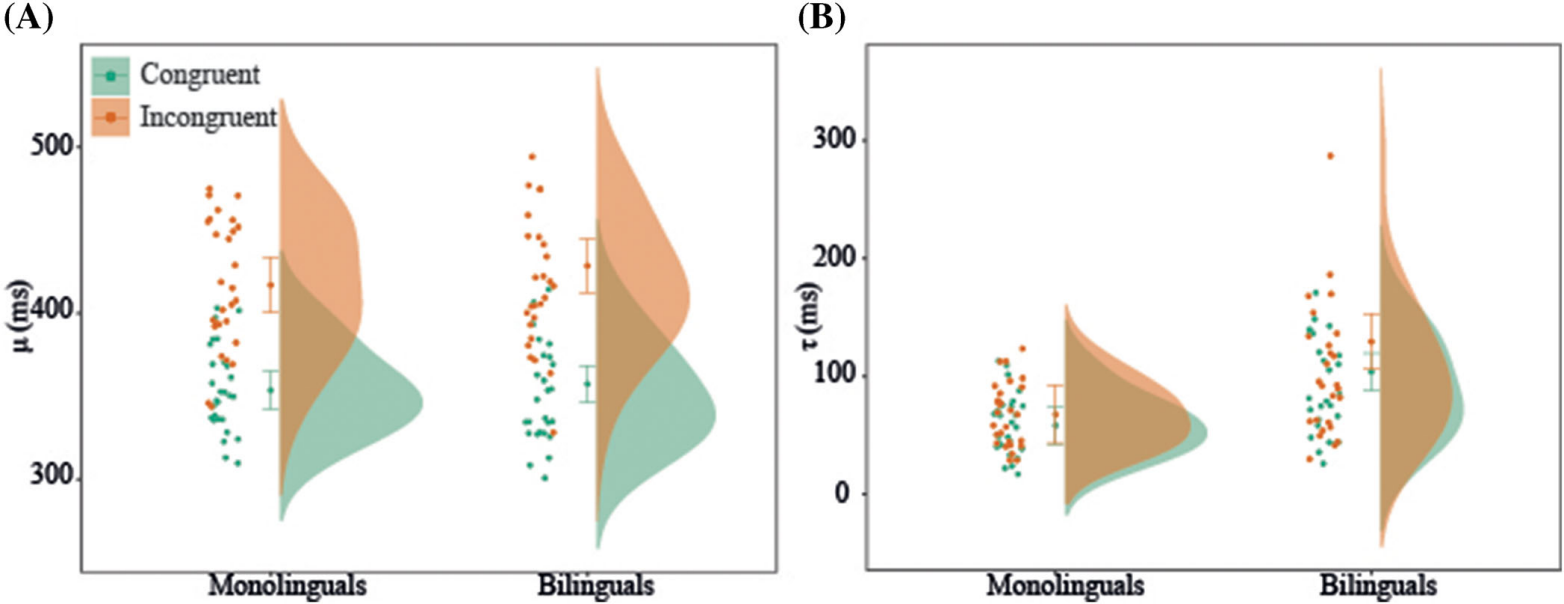
Distributions and means of ex‐Gaussian parameter *μ* (panel A) and ex‐Gaussian parameter τ (panel B) per condition (congruent and incongruent) in the flanker task for both monolinguals and bilinguals. Error bars represent 95% confidence intervals

Figure 1 shows the distributions of average ex‐Gaussian parameters *μ* (panel A) and τ (panel B) of the flanker task for the two participant groups and the two conditions (see Figure S1 for the distributions of average RT and accuracy and Table S1 for the mean and standard deviation of all behavioural parameters).

#### 5.1.1 Ex‐Gaussian μ

There was a significant main effect of Condition, *F*(1,52) = 414.74, *p* < .001, indicating larger *μ* in the incongruent compared to the congruent condition. There was no main effect of Language Group, *F*(1,52) = .035, *p = .852*, nor a Language Group by Condition interaction, *F*(1,52) = .29, *p = .593*.

#### 5.1.2 Ex‐Gaussian *τ*

There was a significant main effect of Condition, *F*(1,52) = 7.16, *p = .010*, indicating larger τ (i.e. longer distribution tails) in the incongruent compared to the congruent condition. There was also a significant main effect of Language Group, *F*(1,52) = 12.19, *p = .001*, with bilinguals having overall larger τ compared to monolinguals. The interaction between Condition and Language Group was not significant, *F*(1,52) = 2.94, *p = .590*.

## 6 EEG RESULTS

### 6.1 ERP condition differences

The non‐parametric cluster‐based permutation tests (with a time window of interest of 0 to 1 s) were performed to investigate ERP differences between congruent and incongruent trials. As the interference effect between congruent and incongruent conditions is well documented, we only briefly outline the results from the cluster‐based permutation tests here as a sanity check (also see Figure S2). The tests revealed significant differences in the ERP waveform in time periods corresponding to the N2 and P3 (the components of interest), as found previously (e.g. Bartholow et al., 2005; Purmann et al., 2011; Wild‐Wall et al., 2008; Yeung et al., 2004). The tests also revealed a condition related late negativity component, which was unexpected however, similar to Jiao et al. (2020).

We observed a significant condition difference (*p = .004*), with the incongruent condition leading to more pronounced negative amplitudes compared to the congruent condition. This corresponded to a cluster ~200 to 400 ms post stimulus presentation and was maximal over central sites. This reflected a condition difference at the N2, maximal between ~300 and 380 ms. Second, there was also a significant P3 difference (*p < .001*), with the incongruent condition leading to a bigger P3 component compared to the congruent condition. This effect was reflected in a cluster spanning from 390 to 790 ms and was maximal over central electrodes. Third, we found a significant condition difference (*p = .004*) with the incongruent condition leading to a greater late negativity (compared to congruent trials). This corresponded to a cluster from 840 to 1000 ms, maximal over central (and posterior) sites.

### 6.2 ERP language group differences

**FIGURE 2:**
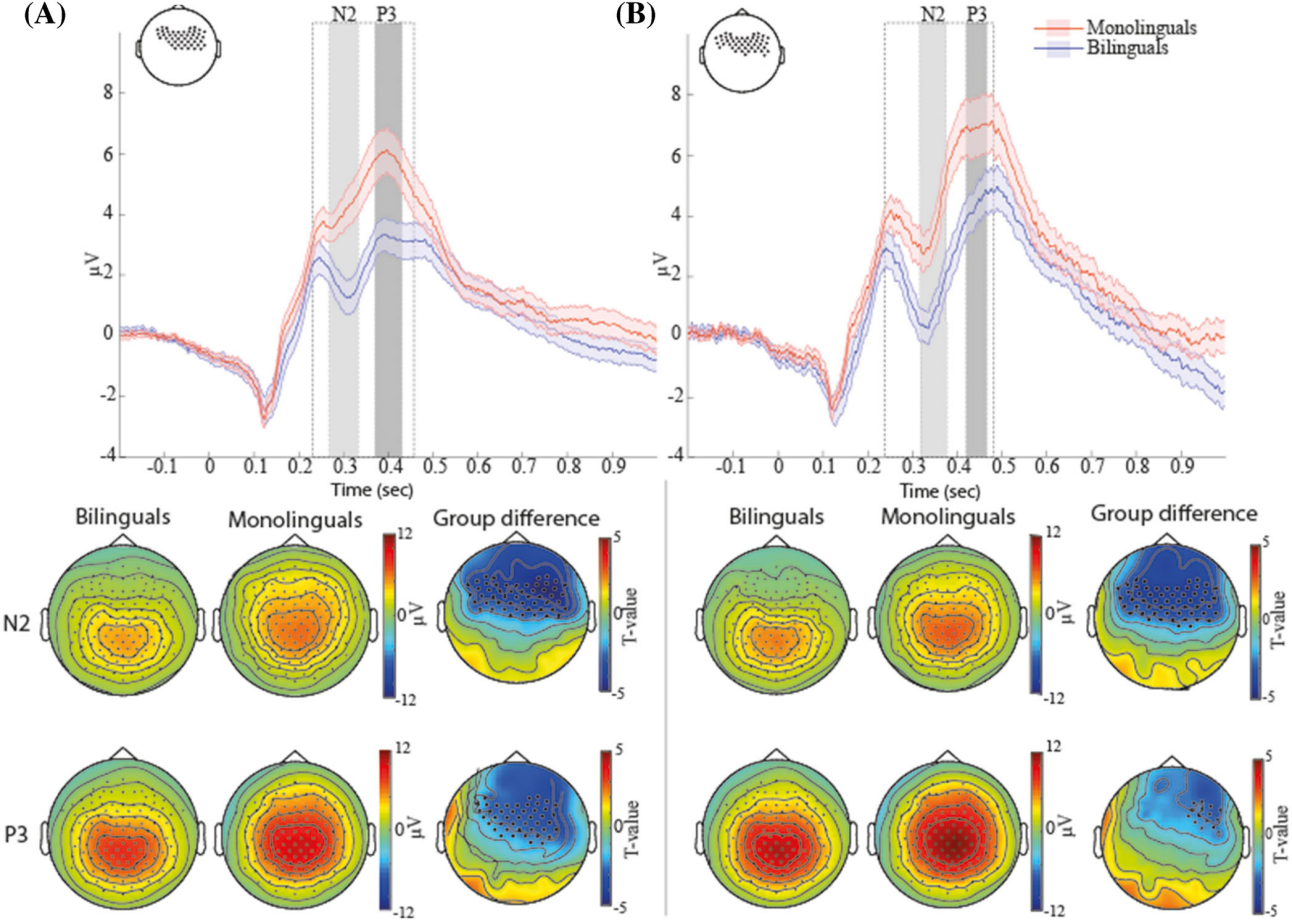
Stimulus locked averaged ERPs produced by (A) congruent trials and (B) incongruent trials in the flanker task for monolinguals (red) and bilinguals (blue). The shaded areas around the ERP waves represent standard error (see Figure S3 for individual ERP waveplots). The dotted rectangles represent the time windows of the significant between‐group differences for each condition. The ERP waveforms show averaged ERPs across the electrode clusters that indicate the maximal group difference (a schematic view of these electrodes is shown in the top left corner of each waveform plot) in the specific time interval (this is for illustrative purposes only, the whole time window of 0 to 1 s was used in the non‐parametric cluster‐based permutation tests). The black dots in the topographic head plots illustrate the cluster of electrodes showing most pronounced mean group differences for the N2 and P3 components. The time windows for N2 and P3 components are shaded in grey and were defined based on visual inspection

**FIGURE 3:**
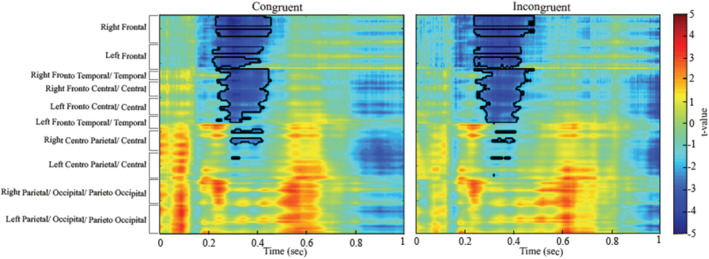
Between language group statistics (*t*‐values) from the non‐parametric cluster‐based permutation tests in the congruent (left) and incongruent (right) conditions with electrode regions on the *y* axis and point‐by‐point time on the *x* axis. The black line contours highlight significant language group differences (as determined through the Monte Carlo cluster‐based permutation tests)

Figure 2 shows stimulus locked averaged ERPs for monolinguals and bilinguals for each condition (congruent and incongruent) as well as topographic distributions of differences (also see Figure S3 for individual ERP waveplots). Figure 3 shows the results of the non‐parametric cluster‐based permutation tests of group differences (monolinguals vs. bilinguals) for each condition (congruent and incongruent conditions) across all electrodes and time. In both conditions, bilinguals had a more pronounced N2 (i.e. a greater negative peak) and a less pronounced P3 than monolinguals. In the congruent condition, the cluster‐based permutation test indicated that there was a significant effect of language group (*p = .018*). We observed a group difference in a time interval encompassing the N2 and P3 components, extending from 220 to 460 ms. Within this time window, the N2 component (defined upon visual inspection between 275 and 325 ms) was maximal over fronto‐central sites, and the P3 component (defined upon visual inspection between 375 and 425 ms) was maximal over fronto‐central and right centro‐parietal electrodes. The N2 was more pronounced (i.e. more pronounced negative‐going amplitudes) in bilinguals compared to monolinguals, and the P3 was smaller in bilinguals compared to monolinguals. In the incongruent condition, the cluster‐based permutation test again indicated that there was a significant effect of language group (*p = .014*). We observed a significant difference in the ERP waveform from around 240 to 480 ms. Within this time window, the N2 component (defined upon visual inspection between 325 and 375 ms) was maximal over fronto‐central sites, and the P3 component (defined upon visual inspection between 425 and 475 ms) was maximal over right fronto‐temporal sites.

**FIGURE 4:**
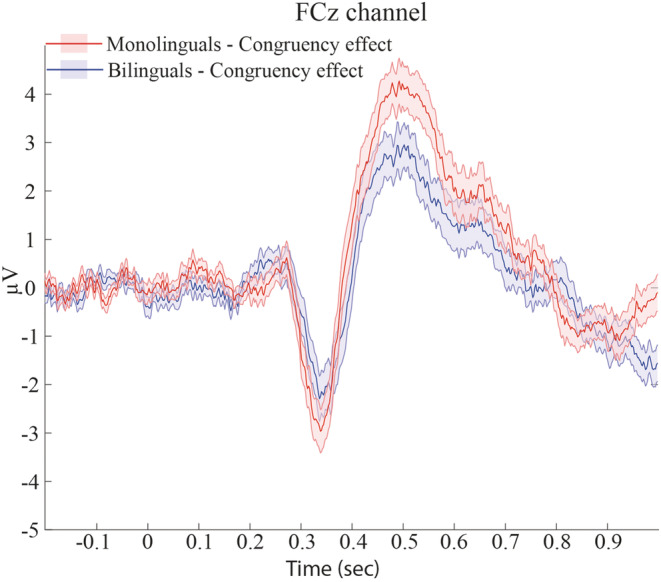
Stimulus locked averaged ERP congruency effect (incongruent trials–congruent trials) for bilinguals (blue) and monolinguals (red) at an exemplary electrode FCz. The shaded areas around the ERP waves represent standard error. Although monolinguals appear to have a larger congruency effect at the P3 component than bilinguals, this difference was not significant

Figure 4 shows the congruency effects (incongruent condition–congruent condition) for both participant groups. Although the figure suggests a smaller congruency effect for P3 amplitudes for bilinguals, the cluster‐based permutation tests did not reveal any significant group differences.

### 6.3 Relationships between behavioural data and ERP amplitudes

We used a data driven approach for calculating the N2 and P3 mean peak amplitudes. The time windows of the N2 and P3 components were defined upon visual inspection of the ERP data where significant differences were observed in cluster‐based permutation tests. The time window used for calculating the mean peak amplitude of the N2 component was 275 to 325 ms for the congruent condition and 325 to 375 ms for the incongruent condition (see also Figure 2). The time window used for calculating the mean peak amplitude of the P3 component was 375 to 425 ms for the congruent condition and 425 to 475 ms for the incongruent condition (see also Figure 2). The electrodes used for calculating the mean peak amplitudes map onto the electrode clusters that revealed significant group differences in the N2 and P3 for congruent and incongruent conditions (marked as black dots in the topographic plots in Figure 2).

**FIGURE 5:**
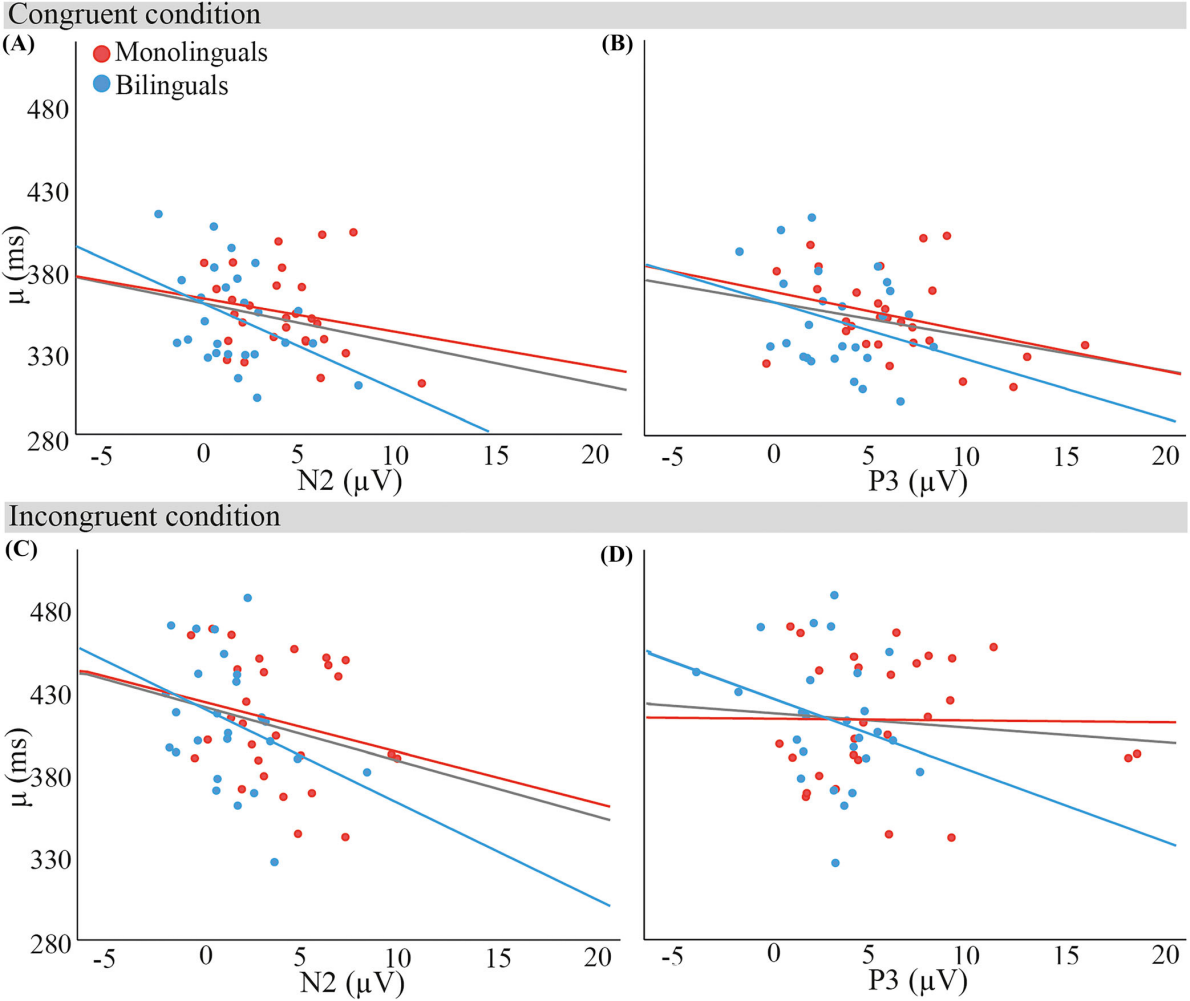
Relationships of *μ* (ms) with mean N2 and P3 peak amplitudes (μV) in the congruent condition (panels A and B) and in the incongruent condition (panels C and D), collapsed over participant groups (*N* = 54). Monolingual data are marked in blue, bilingual data in red. All lines of best fit are least squares regression lines; the grey line reflects all data points, the red line represents bilinguals, and the blue line represents monolinguals. Regression analyses with both neural markers and their interactions with language group showed that neither N2 nor P3 amplitudes predicted *μ* in either condition, irrespective of language group

**FIGURE 6:**
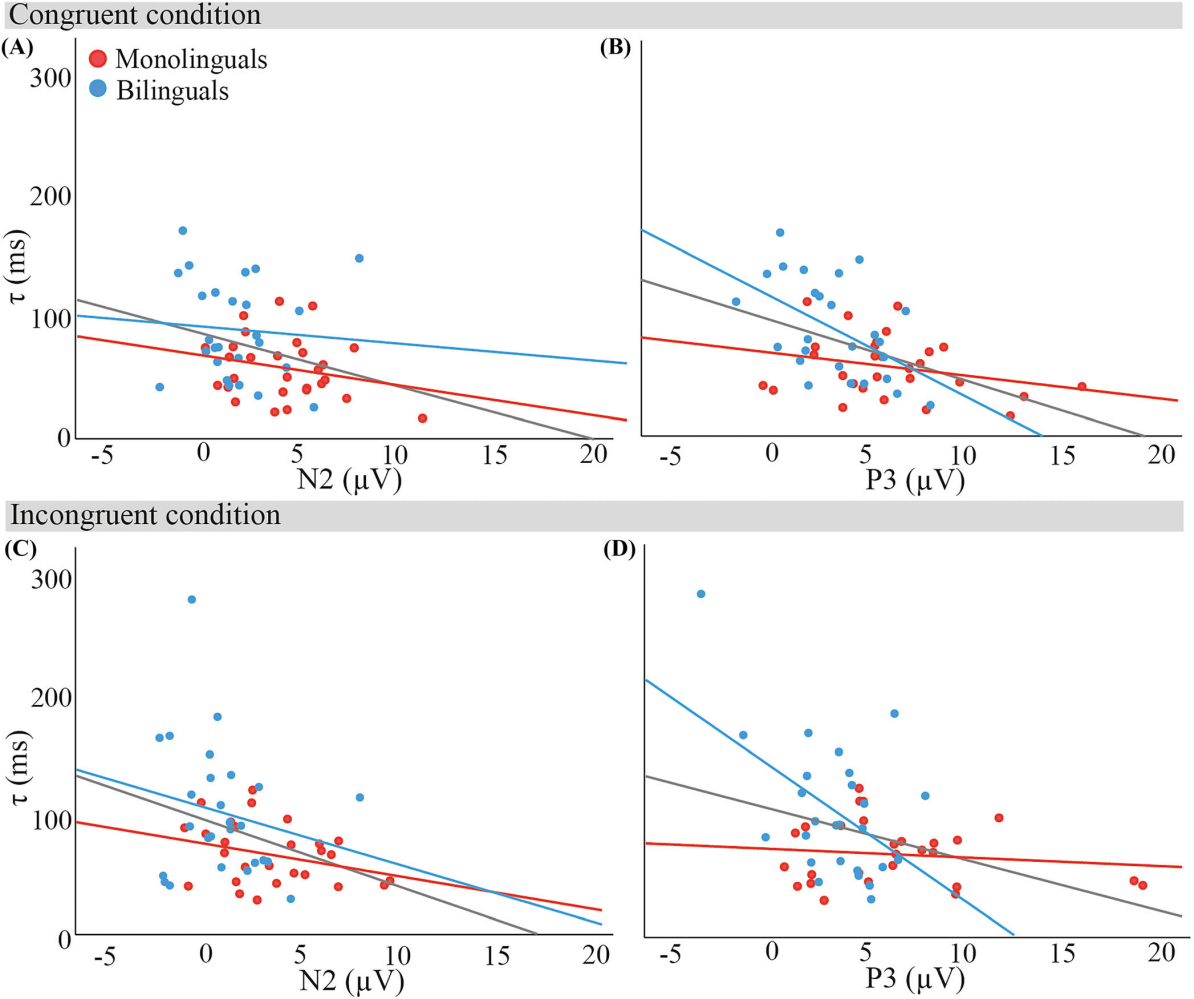
Relationships of τ (ms) with mean N2 and P3 amplitudes (μV) in the congruent condition (panels A and B) and in the incongruent condition (panels C and D), collapsed over participant groups (*N* = 54). Monolingual data are marked in blue, bilingual data in red. All lines of best fit are least squares regression lines; the grey line of best fit reflects all data points, the red line represents bilinguals, and the blue line represents monolinguals. Regression analyses with both neural markers and their interactions with language group showed that for bilingual participants, P3 amplitudes significantly predicted τ in both conditions, whereas there was a trend for N2 amplitudes predicting τ in the congruent condition. There were no significant predictors for monolinguals' τ

Figures 5 and 6 show the relationships of *μ* and τ with N2 and P3 amplitudes for the congruent and incongruent conditions. For both conditions, standardising factors before conducting a regression analysis meant that VIFs of predictors were all below 10 (congruent condition: N2: 5.5, P3: 6.6, Group: 1.5, N2 x Group: 4.6, P3 x Group: 5.8) (incongruent condition: N2: 5.3, P3: 6.3, Group: 2.7, N2 x Group: 8.6, P3 x Group: 6.7).

Table 3 lists the results of the regression analyses for the predictors of ex‐Gaussian *μ* and τ, for both congruent and incongruent conditions. We found that neither N2 nor P3 predicted *μ*, and that this was independent of language group. In contrast to *μ*, P3 amplitudes significantly predicted τ, but we also found a significant Group x P3 interaction. Follow‐up regression analyses in each group showed that τ was significantly predicted by P3 amplitudes only for bilingual participants, but in both congruent and incongruent conditions (see Table 4). In addition, there was a trend for a significant prediction by N2 amplitudes for bilingual participants in the congruent condition.

**TABLE 3:** Results of regression analyses of *μ* and τ in congruent and incongruent conditions

		Congruent		Incongruent	
	Predictors	*t*	*p*	*t*	*t*
*μ*	N2	−.97	.336	−.72	.473
	P3	−.65	.516	−.99	.329
	Group	1.60	.116	1.36	.180
	Group x N2	.79	.432	−.62	.537
	Group x P3	−.021	.831	1.48	.145
τ	N2	2.21	.032	.75	.459
	P3	**3.72**	**<.001**	**−3.55**	**<.001**
	Group	−2.21	.039	−.24	.182
	Group x N2	−1.94	.058	−1.26	.215
	Group x P3	**2.71**	**.009**	**3.18**	**.003**

*Note*: Values in bold indicate significant effects at *α* = .0125.

**TABLE 4:** Results of follow‐up regression analyses, predicting τ in each participant group, for both congruent and incongruent conditions

		Congruent			Incongruent		
	Predictor	VIF	*t*	*p*	VIF	*t*	*p*
Bilinguals	N2	1.5	1.92	.068	1.3	.59	.563
	P3	**1.5**	**−3.22**	**.003**	**1.3**	**−2.80**	**.010**
Monolinguals	N2	1.6	−.45	.656	2.1	1.57	.128
	P3	1.6	−.86	.399	2.1	.69	.496

*Note*: Values in bold indicate significant effects at *α* = .05.

## 7 DISCUSSION

In the present study, we aimed to investigate differences in engagement of sub‐processes of cognitive control between monolingual and bilingual speakers and how these might lead to behavioural differences. The effect of bilingualism was examined on behavioural outcome measures and neural ERP responses in a flanker task. We focussed on the language group differences in the N2 and P3 component amplitudes for each condition separately (congruent and incongruent), as well as on the congruency effect in the neural ERP responses. We replicated what had previously been observed with regard to general behavioural and neural differences for congruent and incongruent conditions in the flanker task. We also found that behaviourally bilinguals had longer response distribution tails than monolinguals, independent of flanker condition (reflected also in a trend towards overall slower RTs in bilinguals, see supporting information). Furthermore, bilinguals exhibited more pronounced N2 and smaller P3 ERP components compared to monolinguals, independent of condition. We did not find any group differences in terms of congruency effects, neither for behavioural nor for neural responses. Furthermore, P3 amplitudes and, to some degree, N2 components predicted response distribution tails for bilingual participants, with smaller P3 amplitudes and larger N2 amplitudes leading to longer tails. But neural markers did not predict the Gaussian part of the response distributions (i.e. the majority of responses).

### 7.1 Behavioural data

We examined ex‐Gaussian parameters *μ* and τ of the RT distributions to assess differences in automatic and controlled processes. In line with previous literature (Heathcote et al., 1991; Spieler et al., 1996; Zhou & Krott, 2018), incongruent trials produced larger *μ* compared to congruent trials (in both language groups), reflecting an anticipated positive shift in response time distribution in the more demanding condition. Similar to the increase in averaged RT, the increased *μ* corresponds with the additional processing cost associated with resolving conflict and active inhibitory mechanisms in the incongruent condition (Zhou & Krott, 2018). Furthermore, the incongruent condition showed larger τ compared to the congruent condition (regardless of language group). The τ parameter has been shown to be less affected by condition differences in conflict tasks (Aarts et al., 2009; Heathcote et al., 1991; Spieler et al., 2000), but a condition difference in τ was found in, for instance, a flanker task by Zhou and Krott (2018). The τ parameter is thought to reflect controlled processes. As discussed, the flanker task is thought to engage various controlled processes: conflict monitoring, the deliberate processing route that involves the stimulus–response translation, and control processes needed to merge the outputs of the two processing routes. These processes are more active or might take more time in the incongruent condition. Thus, if they are not as efficient for some incongruent trials, they can occasionally lead to extremely slow responses.

Bilinguals and monolinguals did not differ in terms of the bulk of the responses (reflected by *μ*), suggesting generally similar automatic processing and similar efficiency in response conflict resolution across the groups (Zhou & Krott, 2018). However, bilinguals exhibited longer response time distribution tails than monolinguals, independent of condition, meaning they occasionally had particularly long responses (reflected in τ). Longer response distribution tails were reflected in a trend for overall slower responses in bilinguals compared to monolinguals. As τ could reflect various controlled processes (conflict monitoring, the deliberate processing route, and the merging of the two processing routes), group differences can arise from differences in one or more of these processes. To determine what these differences might be, it is useful to take into account group differences in neural patterns and how behavioural measures relate to them.

Note that the finding of longer response distribution tails in bilinguals is contrary to previous findings (Abutalebi et al., 2015; Calabria et al., 2011; Tse & Altarriba, 2012; Zhou & Krott, 2018). It is not clear how these differences occur. But again, differences in experimental paradigms (inclusion of a neutral condition, cues preceding the stimulus arrays, carry‐over effects from other tasks and different tasks [Stroop instead of flanker task]) might lead to variations in the engagement of different controlled processes.

### 7.2 Event related brain potential (ERP) data

In line with previous studies (Kousaie & Phillips, 2012, 2017; Purmann et al., 2011; van Veen & Carter, 2002; Wild‐Wall et al., 2008), greater conflict monitoring demands emerging from the incongruent trials led to a significantly larger N2 component compared to the congruent condition (regardless of language group) over central sites. In line with previous results for a very similar version of the flanker task, the N2 peaked between 320 and 380 ms (Yeung et al., 2004). We also observed an unexpected late negativity for incongruent trials from 840 ms. Jiao et al. (2020) also reported such an effect, but they did not interpret it. Importantly, this effect occurred well after responses were given. It therefore most likely reflects either retrospective monitoring processes or the increase of alertness after a challenging incongruent trial. The hypothesis of retrospective monitoring is in line with its distribution, which mirrors that of the N2.

With regards to language group differences, the N2 was more pronounced in bilingual compared to monolingual participants for both congruent and incongruent trials. In contrast, the P3 was significantly reduced for bilinguals compared to monolinguals, again independent of condition. The N2 component in a flanker task has been shown to be sensitive to monitoring demand (Clayson & Larson, 2011; Danielmeier et al., 2009; Folstein & Van Petten, 2008; Grützmann et al., 2014; Purmann et al., 2011; Yeung & Cohen, 2006), suggesting that it reflects strategic proactive control (Bugg & Gonthier, 2020). Our pattern of ERP results therefore suggests that bilinguals allocated greater resources to conflict monitoring than monolinguals, independent of condition. We speculate that bilinguals thus utilised a more proactive processing approach than monolinguals (Bartholow et al., 2005; DeLuca et al., 2020; Grundy, Anderson, & Bialystok, 2017; Tillman & Wiens, 2011). As a consequence of the proactive approach of bilinguals, fewer attentional resources were devoted for conflict control and categorisation, reflected in the smaller fronto‐central P3 component in bilinguals compared to monolinguals. Thus, bilinguals devoted fewer attentional resources to the merging of the two processing routes (direct route and deliberate route).

The group effect on P3 was more frontal than the P3 effect caused by the incongruent flankers and group effects reported in the literature for other types of conflict task (Kousaie & Phillips, 2012, 2017; Moreno et al., 2014). The frontal distribution might indicate a stronger contribution of frontal attentional mechanisms. A smaller P3 component for bilingual speakers might therefore reflect a reduced engagement of frontal attentional processes during stimulus categorisation, in line with the suggestion that L2 usage leads to less reliance on frontal cortical and greater reliance on posterior/subcortical brain regions during cognitive control tasks (DeLuca et al., 2020; Grundy, Anderson, & Bialystok, 2017).

Our results are compatible with various findings for conflict tasks, for example, the findings for Go/No Go tasks, in which bilinguals had more pronounced N2 amplitudes compared to monolinguals in the No Go condition, despite the lack of behavioural performance differences (Fernandez et al., 2013, 2014; Moreno et al., 2014), and in which higher L2 proficiency has been found to lead to larger N2 amplitudes (Fernandez et al., 2013, 2014). The combination of increased N2 and reduced P3 in our study is equivalent to what Jiao et al. (2020) had found for flanker trials embedded in a mixed language context, thus in a situation of enhanced executive control demand. Similarly, a reduced P3 in flanker trials had been reported for a mixed compared to a single language condition (Wu & Thierry, 2013). Furthermore, bilingual speakers have shown reduced P3 amplitudes compared to monolingual speakers in a flanker task (Botezatu et al., 2021) previously, as well as in a Stroop task (Coderre et al., 2014) and a Simon task (Kousaie & Phillips, 2012). These results all suggest that bilinguals allocate less attentional resources to the merging of competing responses and categorisation processes than monolingual speakers in a conflict task.

In contrast, Kousaie and Phillips (2012, 2017) did not find language group differences in N2 or P3 amplitudes in a flanker task. Instead, they discovered the opposite N2 effect in a verbal Stroop task, with monolinguals showing a larger N2 than bilinguals. As noted, the results for the verbal Stroop task might be because of more resources being diverted to linguistic processes during the task. Finally, bilinguals showed greater instead of reduced P3 amplitudes in Stroop and Simon tasks. One remarkable difference between these two studies and the current study is that they included not only congruent and incongruent trials but also 33% neutral trials. Since both N2 and P3 are very sensitive to executive function demands of a task, especially monitoring demands (Jiao et al., 2020; Purmann et al., 2011; Wu & Thierry, 2013), it is possible that the inclusion of neutral trials shifted the balance of monitoring and attention to conflict resolution processes in the two participant groups. Although the proportion of incongruent trials (25% in the current version and 33% in Kousaie and Philips' version) is quite similar, the participants had to only monitor for incongruent flankers in Kousaie and Philips' studies when a stimulus actually included flankers (i.e. for incongruent and congruent trials). This might have changed bilinguals' proactive monitoring approach to a more reactive approach, thus one very similar to that of monolingual participants. This also explains that Botezatu et al. (2021) did not find an N2 group difference when using the same proportion of trials as Kousaie and Philips.

Proactive control is more effortful but known to be generally more effective than reactive control (Braver, 2012), and therefore usually advantageous. However, bilinguals' proactive enhanced resource allocation to monitoring in the present study was not quite advantageous. Their enhanced N2 amplitudes were related to smaller P3 amplitudes, which in turn were related to longer distribution tails. And there was a trend for larger N2 amplitudes being related to longer distribution tails as well (albeit only in the congruent condition). Thus, enhanced bilingual monitoring was associated with reduced attentional resources allocated likely to the merging of the two processing routes (direct route and deliberate route) in the task. The latter seems to have occasionally led to very slow responses. Stronger engagement of monitoring processes might mean that responses were checked particularly thoroughly, for instance, by increasing a response activation threshold. This would mean that bilinguals slightly over‐engaged in monitoring in the current study. As mentioned, the relatively rare (25%) occurrence of incongruent trials might have activated the generally proactive monitoring approach in bilinguals, whereas monolinguals took a more reactive processing approach, relying more strongly on resource allocation to resolving any response conflict. The latter might have been slightly more beneficial at times, as evidenced in fewer extremely long responses in the monolingual group. In other words, bilinguals' enhanced monitoring was generally balanced by more efficient conflict resolution and categorisation (reflected in reduced P3 amplitudes) in the present task, apart from some occasions where the latter led to extremely long responses.

Furthermore, the relationship between neural markers (N2, P3) and behavioural measures (ex‐Gaussian parameters) were affected by participant group membership. The P3 was related to response distribution tails exclusively for bilinguals, with an additional trend for a relationship with N2 for the congruent condition. Thus, the up‐ and down‐regulating of attentional resources for conflict processing seems to be linked to the occurrence of long responses in bilinguals only. It is not clear why this might be the case. As regressions can be affected by sample size, future studies will need to investigate whether this can be replicated with a larger participant group.

Overall, we found evidence that stronger engagement of sub‐processes in the task, such as devotion of attention to conflict resolution (P3), is beneficial for more controlled processing (ex‐Gaussian parameter τ) in the flanker task. We did not find evidence for such a beneficial relationship with more automatic processing (ex‐Gaussian parameter *μ*).

### 7.3 The bilingual advantage in conflict tasks

What do the current findings mean with regard to the debate about the bilingual advantage in conflict tasks? The current literature is equivocal. Specifically in the flanker task, some previous studies have found that bilinguals are faster (Costa et al., 2008; Emmorey et al., 2008) and more accurate (Kousaie & Phillips, 2017) than monolinguals. Other studies have reported no significant RT differences between bilinguals and monolinguals (Antón et al., 2019; Grundy, Chung‐Fat‐Yim, et al., 2017; Kousaie & Phillips, 2012, 2017; Luk et al., 2010) or, similarly to our study, a small non‐significant bilingual disadvantage (Paap & Greenberg, 2013; Paap & Sawi, 2014). Taken together, there is no consistent advantage in flanker tasks or, more generally, in conflict tasks (see Ware et al., 2020, who found that different tasks are more or less consistent in showing a bilingual advantage). An advantage likely only appears in conditions with the right balance of demands on sub‐processes. If proactive monitoring is advantageous in a paradigm, bilinguals might process the conflict more efficiently and might therefore be more likely to show a behavioural advantage. However, proactive conflict monitoring does not always lead to faster (or more accurate) responses. As evident in the current study, it can occasionally backfire and hinder performance. In other words, although it is not possible to pinpoint one particular difference that explains the inconsistencies across studies, differences in conflict monitoring demands across studies (because of, for example, carry‐over effects from other tasks; e.g. Antón et al., 2019) or methodological differences such as additional neutral trials (Kousaie & Phillips, 2012, 2017) might be one of the reasons.

## 8 INDIVIDUAL DIFFERENCES

Although group differences in conflict tasks can be affected by task parameters, performance of bilinguals is also impacted by individual variations in bilingual experience. Language experiences such as intensity and diversity of L1 and L2 use, language switching in daily life, relative proficiency and duration of the two languages have varying consequences on control demands and lead to both structural and functional neural changes (see the Unifying the Bilingual Experience Trajectories [UBET] model by DeLuca et al., 2020). For instance, models of individual variation of bilingual experience onto structural and functional brain changes predict less reliance on frontal areas with longer L2 use (DeLuca et al., 2020; Grundy, Anderson, & Bialystok, 2017). Not surprisingly, there are increasing calls to account for individual differences in bilingual experiences in the bilingual advantage literature (Poarch & Krott, 2019). The effect of such variations has indeed been shown in both the flanker task (Dong & Zhong, 2017; Hofweber et al., 2016) and in other tasks with a suppression component (Fernandez et al., 2013, 2014; Gullifer et al., 2018; Sullivan et al., 2014). For the flanker task, bilinguals who engage in more dense code‐switching have shown inhibitory advantages in a condition with medium monitoring demand (25% incongruent trials) (Hofweber et al., 2016); and interpreting experience has been shown to heighten early attentional processing (larger N1), conflict monitoring (larger N2) and interference suppression (smaller P3 and smaller RT interference effects) (Dong & Zhong, 2017). L2 proficiency seems to particularly impact neural markers in a Go/No Go task, with higher proficiency leading to more pronounced N2 (Fernandez et al., 2013) and a 6‐month University Spanish course (compared to a Psychology course) increasing the P3 component (Sullivan et al., 2014). Furthermore, proactive and reactive control can be affected by different experiences. Gullifer et al. (2018) reported that greater diversity in language use in daily life was related with greater reliance on proactive control in an AX‐Continuous performance task, whereas earlier L2 age of acquisition was associated with a decrease in reliance on proactive control. Future studies will therefore need to take into account not only task parameters but also individual variations in bilingual experience.

To conclude, we have found that bilingual speakers showed evidence for enhanced proactive monitoring during a flanker task with medium monitoring demand compared to monolingual speakers. We speculate that the enhanced monitoring was followed by less anterior attentional resources devoted to conflict resolution and stimulus categorisation, thus by less effortful categorisation. Reduced anterior attentional engagement, however, led occasionally to very slow responses. Thus, we speculatively propose that bilingual enhanced monitoring and reduced attentional resources for categorisation more or less balanced each other out, but reduced anterior resources for categorisation slightly dominated. These results demonstrate how the engagement of sub‐processes of a task together determines overall behavioural performance and can affect group differences. Although there is currently a strong focus shift in the literature on how individual differences in bilingual experience might affect bilingual performance and therefore differences compared to monolingual speakers, we propose that the study of balance of sub‐processes in conflict tasks is a complementary avenue that is useful for a better understanding of any functional differences between bilingual and monolingual speakers.

## CONFLICTS OF INTEREST

The authors declare no conflicts of interest.

## AUTHOR CONTRIBUTIONS

AK conceptualised the study. RM collected the data and analysed the data. AK and AM supervised data analyses. RM wrote the manuscript. AK supported the manuscript writing. All authors edited the manuscript.

### PEER REVIEW

The peer review history for this article is available at https://publons.com/publon/10.1111/ejn.15863.

## Supporting information


**Figure S1.** Distributions and means of RT (panel A), and accuracy (panel B) per condition (congruent and incongruent) in the flanker task for both monolinguals and bilinguals. Error bars represent 95% confidence intervals.
***RT.*** We found a significant main effect of Condition, *F*(1,52) = 405.32, *p < .001*, indicating overall slower RTs in the incongruent compared to the congruent condition. There was a trend for a main effect of Language Group, *F*(1,52) = 3.53, *p = .066*, with bilinguals tending to have overall longer RTs compared to monolinguals. The interaction between Condition and Language Group was not significant, *F*(1,52) = .98, *p = .327*.
***Accuracy.*** There was a significant main effect of Condition, *F*(1,52) = 142.95, *p < .001,* indicating overall lower accuracy in the incongruent compared to the congruent condition. There was no main effect of Language Group, *F*(1,52) = .22, *p = .642,* nor a Language Group by Condition interaction: *F*(1,52) = .34, *p = .564*.

**Figure S2.** Stimulus locked averaged ERPs for congruent trials (green), incongruent trials (orange) and the congruency effect (black) in the flanker task, averaged across all participants at the FCz channel. The shading around the ERP waves represents standard error. The dotted rectangles represent the time windows of the three significant between‐condition differences (i.e., the N2, P3, and late negativity). The head plots illustrate the topographic distribution of these differences, averaged over the respective significant time window (note that the grey shading is purely for illustrative purposes centred around the N2 (300‐380ms) and P3 (410‐550ms) peaks with the topographic distribution matching the respective time windows). The topographic distribution of the late negativity component is averaged over the time window marked by the dotted rectangle.

**Figure S3.** Stimulus locked individual ERPs produced by congruent (left) and incongruent (right) trials in the flanker task for monolinguals (red) and bilinguals (blue). The ERP waveforms show averaged ERPs across the electrode clusters that indicate the maximal group difference (a schematic view of these electrodes is shown in the top left corner of each waveform plot). Each ERP wave represents one participant.

**Table S1.** Mean and standard deviation of behavioural parameters presented in Figure 1 and Figure S1.

## Data Availability

Stimuli and data are available upon request.
