# Supplementary figures and images for: Bilingualism can cause enhanced monitoring and occasional delayed responses in a flanker task
Source: Eur J Neurosci. 2022 Nov 23;57(1):129–47. doi: 10.1111/ejn.15863 (PMC10100525; doi:10.1111/ejn.15863)

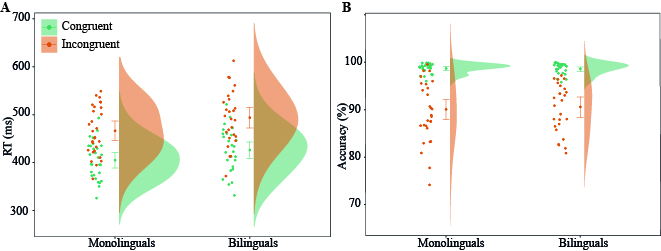

Supplement: Supplementary file 1 — Figure S1. Distributions and means of RT (panel A), and accuracy (panel B) per condition (congruent and incongruent) in the flanker task for both monolinguals and bilinguals. Error bars represent 95% confidence intervals. RT. We found a significant main effect of Condition, F(1,52) = 405.32, p < .001, indicating overall slower RTs in the incongruent compared to the congruent condition. There was a trend for a main effect of Language Group, F(1,52) = 3.53, p = .066, with bilinguals tending to have overall longer RTs compared to monolinguals. The interaction between Condition and Language Group was not significant, F(1,52) = .98, p = .327. Accuracy. There was a significant main effect of Condition, F(1,52) = 142.95, p < .001, indicating overall lower accuracy in the incongruent compared to the congruent condition. There was no main effect of Language Group, F(1,52) = .22, p = .642, nor a Language Group by Condition interaction: F(1,52) = .34, p = .564. [file EJN-57-129-s004.jpg]

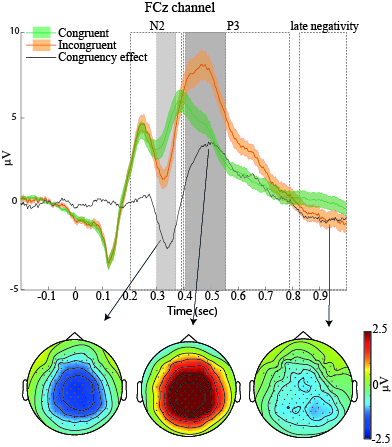

Supplement: Supplementary file 2 — Figure S2. Stimulus locked averaged ERPs for congruent trials (green), incongruent trials (orange) and the congruency effect (black) in the flanker task, averaged across all participants at the FCz channel. The shading around the ERP waves represents standard error. The dotted rectangles represent the time windows of the three significant between‐condition differences (i.e., the N2, P3, and late negativity). The head plots illustrate the topographic distribution of these differences, averaged over the respective significant time window (note that the grey shading is purely for illustrative purposes centred around the N2 (300‐380ms) and P3 (410‐550ms) peaks with the topographic distribution matching the respective time windows). The topographic distribution of the late negativity component is averaged over the time window marked by the dotted rectangle. [file EJN-57-129-s002.jpg]

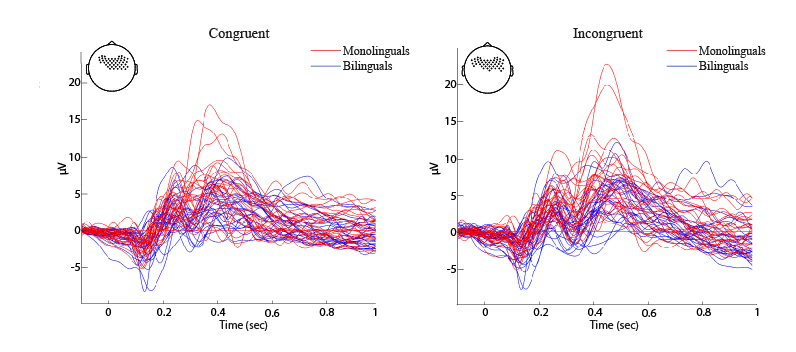

Supplement: Supplementary file 3 — Figure S3. Stimulus locked individual ERPs produced by congruent (left) and incongruent (right) trials in the flanker task for monolinguals (red) and bilinguals (blue). The ERP waveforms show averaged ERPs across the electrode clusters that indicate the maximal group difference (a schematic view of these electrodes is shown in the top left corner of each waveform plot). Each ERP wave represents one participant. [file EJN-57-129-s001.jpg]
