# Supplementary material for: Bilingualism can cause enhanced monitoring and occasional delayed responses in a flanker task
Source: Eur J Neurosci. 2022 Nov 23;57(1):129–47. doi: 10.1111/ejn.15863 (PMC10100525; doi:10.1111/ejn.15863)
Supplement: Supplementary file 4 — Table S1. Mean and standard deviation of behavioural parameters presented in Figure 1 and Figure S1. [file EJN-57-129-s003.docx]

**Supplementary Material**

**Traditional measures (i.e., averaged RTs and accuracy) of bilingual and monolingual groups on the Flanker task**


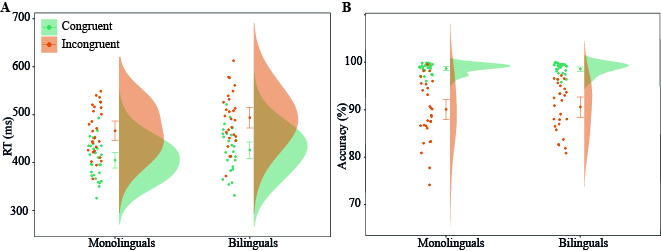


*Supplementary Figure 1.* Distributions and means of RT (panel A), and accuracy (panel B) per condition (congruent and incongruent) in the flanker task for both monolinguals and bilinguals. Error bars represent 95% confidence intervals.

***RT.*** We found a significant main effect of Condition, *F*(1,52) = 405.32, *p < .001*, indicating overall slower RTs in the incongruent compared to the congruent condition. There was a trend for a main effect of Language Group, *F*(1,52) = 3.53, *p = .066*, with bilinguals tending to have overall longer RTs compared to monolinguals. The interaction between Condition and Language Group was not significant, *F*(1,52) = .98, *p = .327*.

***Accuracy.*** There was a significant main effect of Condition, *F*(1,52) = 142.95, *p < .001,* indicating overall lower accuracy in the incongruent compared to the congruent condition. There was no main effect of Language Group, *F*(1,52) = .22, *p =.642,* nor a Language Group by Condition interaction: *F*(1,52) = .34, *p = .564*.


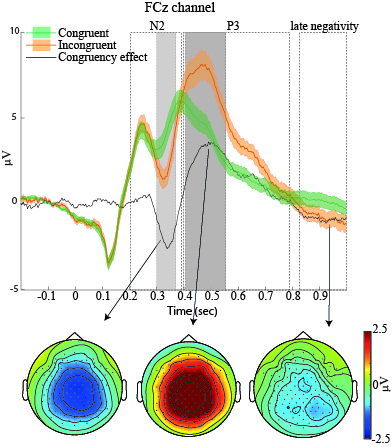


*Supplementary Figure 2.*  Stimulus locked averaged ERPs for congruent trials (green), incongruent trials (orange) and the congruency effect (black) in the flanker task, averaged across all participants at the FCz channel. The shading around the ERP waves represents standard error. The dotted rectangles represent the time windows of the three significant between-condition differences (i.e., the N2, P3, and late negativity). The head plots illustrate the topographic distribution of these differences, averaged over the respective significant time window (note that the grey shading is purely for illustrative purposes centred around the N2 (300-380ms) and P3 (410-550ms) peaks with the topographic distribution matching the respective time windows). The topographic distribution of the late negativity component is averaged over the time window marked by the dotted rectangle.

Supplementary Table 1. *Mean and standard deviation of behavioural parameters presented in Figure 1 and Supplementary Figure 1.*

|  | **RT (ms)**  ***(SD)*** | | **Accuracy (%)**  ***(SD)*** | | **Mu (ms)**  ***(SD)*** | | **Tau (ms)**  ***(SD)*** | |
| --- | --- | --- | --- | --- | --- | --- | --- | --- |
|  | Congruent | Incongruent | Congruent | Incongruent | Congruent | Incongruent | Congruent | Incongruent |
| Monolinguals | 405.04 *(39.62)* | 466.20 *(51.09)* | 98.76 *(1.01)* | 89.36  *(6.38)* | 353.75 *(25.67)* | 416.94 *(38.81)* | 58.50 *(25.44)* | 67.88 *(26.80)* |
| Bilinguals | 426.17 *(46.51)* | 493.65 *(57.18)* | 98.73 *(1.17)* | 89.76  *(5.78)* | 350.41 *(29.94)* | 417.02 *(39.69)* | 90.01 *(40.46)* | 104.15 *(57.02)* |


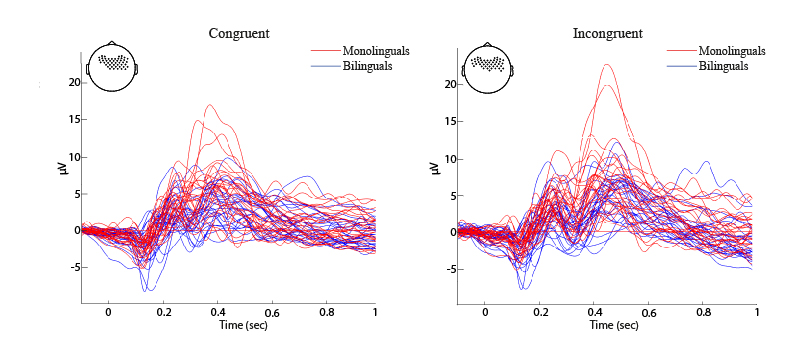


*Supplementary Figure 3.* Stimulus locked individual ERPs produced by congruent (left) and incongruent (right) trials in the flanker task for monolinguals (red) and bilinguals (blue). The ERP waveforms show averaged ERPs across the electrode clusters that indicate the maximal group difference (a schematic view of these electrodes is shown in the top left corner of each waveform plot). Each ERP wave represents one participant.

**Figure captions**

*Supplementary Figure 1.* Distributions and means of RT (panel A), and accuracy (panel B) per condition (congruent and incongruent) in the flanker task for both monolinguals and bilinguals. Error bars represent 95% confidence intervals.

*Supplementary Figure 2.*  Stimulus locked averaged ERPs for congruent trials (green), incongruent trials (orange) and the congruency effect (black) in the flanker task, averaged across all participants at the FCz channel. The shading around the ERP waves represents standard error. The dotted rectangles represent the time windows of the three significant between-condition differences (i.e., the N2, P3, and late negativity). The head plots illustrate the topographic distribution of these differences, averaged over the respective significant time window (note that the grey shading is purely for illustrative purposes centred around the N2 (300-380ms) and P3 (410-550ms) peaks with the topographic distribution matching the respective time windows). The topographic distribution of the late negativity component is averaged over the time window marked by the dotted rectangle.

*Supplementary Figure 3.* Stimulus locked individual ERPs produced by congruent (left) and incongruent (right) trials in the flanker task for monolinguals (red) and bilinguals (blue). The ERP waveforms show averaged ERPs across the electrode clusters that indicate the maximal group difference (a schematic view of these electrodes is shown in the top left corner of each waveform plot). Each ERP wave represents one participant.

**Table captions**

Supplementary Table 1. *Mean and standard deviation of behavioural parameters presented in Figure 1 and Supplementary Figure 1.*
